# Peptic ulcer disease and heart disease are associated with periprosthetic fractures after total hip replacement

**DOI:** 10.3109/17453674.2012.717844

**Published:** 2012-08-25

**Authors:** Jasvinder A Singh, David G Lewallen

**Affiliations:** ^1^Medicine Service and Center for Surgical Medical Acute Care Research and Transitions (C-SMART), Birmingham VA Medical Center, Birmingham, AL; ^2^Department of Medicine at the School of Medicine, and Division of Epidemiology at the School of Public Health, University of Alabama, Birmingham, AL; ^3^Department of Orthopedic Surgery, Mayo Clinic College of Medicine, Rochester, MN, USA.

## Abstract

**Background and purpose:**

There have been no published studies assessing the possible association of medical comorbidities with periprosthetic fracture risk. We therefore assessed whether medical comorbidity is associated with risk of periprosthetic fractures after total hip replacement (THR).

**Material and methods:**

We used prospectively collected data from 1989–2008 in the Mayo Clinic Total Joint Registry for 2 cohorts: primary THR and revision THR. The main variables of interest were Deyo-Charlson comorbidities at the time of surgery. Outcome of interest was p ostoperative periprosthetic fracture at postoperative day 1 onwards. Multivariable

Cox regression models were additionally adjusted for age, sex, body mass index, American Society of Anesthesiology (ASA) class, and operative diagnosis.

**Results:**

We identified 14,065 primary THRs and 6,281 revision THRs with mean follow-up times of 6.3 and 5.6 years, respectively. There were 305 postoperative periprosthetic fractures in the primary THR cohort and 330 in the revision THR cohort. In patients who underwent primary THR, 2 comorbidities were associated with higher risk of periprosthetic fracture: peptic ulcer disease with adjusted hazard ratio of 1.5 (95% CI: 1.1–2.2) and heart disease with adjusted hazard ratio of 1.7 (CI: 1.2–2.4). In patients with revision THR, peptic ulcer disease was associated with a higher adjusted risk of periprosthetic fracture, 1.6 (CI: 1.1–2.3).

**Interpretation:**

Peptic ulcer disease and heart disease in primary THR patients and peptic ulcer disease in revision THR patients were associated with higher postoperative periprosthetic fracture risk. Further studies are needed to understand whether disease severity or specific medications used for treatment, or both, are responsible for this association. This may allow identification of modifiable factors.

Periprosthetic fracture after total hip replacement (THR) is associated with poorer function and poorer quality of life (Young et al. 2008), increased patient morbidity and mortality (Lindahl et al. 2007, Young et al. 2008), and higher use of healthcare and higher costs (Bozic et al. 2005). Despite its substantial effect on patient outcomes and utilization of resources, few registry studies (Lindahl et al. 2005, 2006, Gjertsen et al. 2007) and single-center studies (Wu et al. 1999, Sarvilinna et al. 2004) have examined the factors associated with of periprosthetic fractures after THR. A previous history of fracture (Sarvilinna et al. 2004, Lindahl et al. 2006, Gjertsen et al. 2007), older age (Wu et al. 1999), poorer bone quality (Wu et al. 1999), and Charnley and Exeter implants (Lindahl et al. 2005) have been associated with higher risk of periprosthetic fracture after THR. To our knowledge, none of the previous studies have focused on comorbidity as a risk factor for periprosthetic fractures. A PubMed search using the terms “hip arthroplasty”, “periprosthetic fracture”, and “comorbidity” that was performed in October 2011 found only 3 studies (Lombardi et al. 2007, Pap and Neumann 2007, Zuurmond et al. 2007), and none of them were original articles.

Patients undergoing THR have high comorbidity load (Lubbeke et al. 2007, Singh and Sloan 2009), which is associated with higher hospital costs and utilization of health care resources (Shah et al. 2004), higher implant dislocation rates (Malkani et al. 2010), and higher overall 90-day composite complication rate (Soohoo et al. 2010). As indications for THR broaden to include older patients, knowing which comorbidity is associated with specific post-arthroplasty complications becomes important. It is not known whether certain medical comorbidities increase the risk of periprosthetic fractures after THR. We recently found that peptic ulcer disease and chronic obstructive lung disease were associated with higher risk of postoperative periprosthetic fractures after primary total knee replacement (TKR) (Singh and Lewallen 2011). In this study, we investigated whether common preoperative comorbidities are associated with the risk of postoperative periprosthetic fractures in patients who have undergone primary or revision THR.

## Material and methods

### Study cohort

We identified 2 patient cohorts at the Mayo Clinic, Rochester, MN: those who had undergone primary total hip replacement (primary THR) and those who had undergone revision THR in the period 1989–2008. This time interval was chosen since the databases had prospectively captured information on comorbidity, body mass index (BMI), and American Society of Anesthesiologists (ASA) class) for this period. The Mayo Clinic Total Joint Registry captures demographic, clinical, and implant-related information for each patient undergoing joint replacement surgery at the Mayo Clinic (Berry et al. 1997, Singh et al. 2008). Trained and dedicated members of the registry staff contact each patient prospectively and monitor them for clinically important postoperative outcomes, including revision (Peterson and Lewallen 1996, Lewallen and Berry 1998, Ortiguera and Berry 2002, Parvizi et al. 2004, Alden et al. 2010). For patients who failed to return to the clinic, mailed questionnaires and (when needed) a telephone-based survey is performed by the registry staff, focusing on complications and other important outcomes. Medical records, radiographs, and other relevant data were obtained from other medical facilities for patients who did not return for regular follow-up and/or received care for these complications at other facilities.

The study was approved by the Mayo Clinic Institutional Review Board and all investigations were conducted in conformity with ethical principles of research.

### Outcome and predictor variables and definitions

The outcome of interest was postoperative periprosthetic fracture on postoperative day 1 or later. This period was chosen to identify patients with only postoperative fractures. Intraoperative fractures were not included, since their etiology was thought to be different from that of postoperative fractures. Fractures on postoperative day 0 were not included, as it was difficult to confidently differentiate intraoperative fractures from postoperative ones on that day. Statistical models were used to examine time to first postoperative periprosthetic fracture for each patient under observation.

The main predictors of interest were individual Deyo-Charlson index comorbidities preoperatively (Charlson et al. 1987a, b). Deyo-charlson index is a valid measure of comorbidity. We grouped these comorbidities based on a priori clinical decision as follows, to avoid having too many variables in the analyses: heart disease (myocardial infarction, congestive heart failure); peripheral vascular disease; cerebrovascular disease, hemiplegia or paraplegia; moderate-to severe renal disease; peptic ulcer disease; chronic obstructive pulmonary disease; diabetes (with or without organ damage); connective tissue disease; cancer (leukemia, lymphoma, any other tumor, metastatic solid tumor); and other (dementia, liver disease, AIDS).

### Statistics

We performed separate analyses for the primary THR and revision THR cohorts. We first performed crude (or unadjusted) Cox regression analyses assessing the association of each Charlson comorbidity group (as above) with time to postoperative periprosthetic fracture, separately for primary THR and revision THR. Each patient only contributed 1 observation. The period of observation ended at the time of first postoperative periprosthetic fracture or death, whichever occurred earlier. To assess whether these associations were independent of other factors, we simultaneously adjusted for all the following important variables in multivariable-adjusted Cox regression models (in addition to individual Charlson comorbidity): age (≤ 60, 61–70, 71–80, and > 80 years), sex, body mass index categorized according to the WHO classification (< 25, 25.0–29.9, 30.0–39.9, ≥ 40), American Society of Anesthesiologists (ASA) Physical Status score class (class 1, 2, 3, 4), operative diagnosis (for primary THA-osteoarthritis, rheumatoid arthritis, avascular necrosis, and; for revision THA-loosening/wear/ osteolysis, previous surgery, fracture/dislocation, nonunion, infection, and other) and implant fixation for primary THR only (cemented/hybrid, uncemented). These covariates were included since they were previously known or suspected to be associated with periprosthetic fracture. Potentially correlated variables were tested and were found not to be significantly correlated (defined a priori as < 0.5), and were therefore included in the regression analyses (Charlson index and ASA class, Spearman’s correlation coefficient 0.33). The proportional hazards assumption was tested and held true for the multivariable models. Hazard ratios (HRs) and 95% confidence intervals (CIs) are given. To examine period effect, we performed sensitivity analyses by adjusting multivariable-adjusted Cox regression models for time period (categorized as 1989–94, 1995–99, 2000–04, and 2005–09).

In exploratory analyses, we examined fracture-free survival at 1, 2, 5, and 10 years by the key significant factors associated with postoperative fractures, using the Kaplan-Meier analyses separately by presence and absence of each significant comorbidity.

## Results

There were 14,065 primary THRs and 6,281 revision THRs. The mean ages of the cohorts were 65 years; half were women and three-quarters were overweight or obese. In the primary THR cohort, the most common underlying diagnosis was osteoarthritis (87%) (Table 1). In the revision THR cohort, the commonest underlying diagnosis was loosening/wear/osteolysis (66%). Cemented or hybrid implant fixation was used in 38% of primary THR implants.

**Table 1.** Demographic and clinical features of study cohorts. Values are mean (SD) or n (%)

	Primary THR (n = 14,065)	Revision THR (n = 6,281)
Male/female	6,819/7,246	2,915/3,366
Percentage	(48.5%)/(51.5%)	(46.4%)/(53.6%)
Mean age at surgery in years	64.6 (13.7)	65.3 (13.7)
Age category		
≤ 60 years	4,455 (31.7%)	1,985 (31.6%)
61–70 years	4,252 (30.2%)	1,678 (26.7%)
71–80 years	4,119 (29.3%)	1,932 (30.8%)
> 80 years	1,239 (8.8%)	686 (10.9%)
Mean body mass index (BMI)	29.0 (5.8)	28.4 (5.8)
BMI category, kg/m^a^		
Missing	72 (0.5%)	66 (1%)
Normal, < 25.0	3,429 (24.5%)	1,833 (29.5%)
Overweight, 25.0–29.9	5,334 (38.1%)	2,292 (36.9%)
Obese, 30.0–39.9	4,589 (32.8%)	1,845 (29.7%)
Morbidly obese,≥ 40.0	641 (4.6%)	245 (3.9%)
Unilateral/bilateral	11,772/2,293	5,034/1,247
Percentage	(83.7%)/(16.3%)	(80.1%)/(19.9%)
ASA score**^b^**		
Missing	60 (0.4%)	33 (0.5%)
1	653 (4.7%)	172 (2.8%)
2	7,947 (56.7%)	2,961 (47.4%)
3	5,254 (37.5%)	3,018 (48.3%)
4	151 (1.1%)	97 (1.6%)
Mean Deyo-Charlson index	1.3 (2.2)	1.1 (1.9)
Deyo-Charlson index group		
Heart disease (MI, CHF)	1,211 (8.6%)	542 (8.6%)
Peripheral vascular disease	808 (5.7%)	298 (4.7%)
Cerebrovascular disease, hemiplegia or		
paraplegia	1,125 (8.0%)	432 (6.9%)
Moderate-to-severe renal disease	889 (6.3%)	388 (6.2%)
Peptic ulcer disease	1,031 (7.3%)	435 (6.9%)
Chronic obstructive pulmonary disease	1,568 (11.1%)	564 (9.0%)
Diabetes (with or without organ damage)	1,235 (8.8%)	577 (9.2%)
Connective tissue disease	1,020 (7.3%)	603 (9.6%)
Cancer	2,166 (15.4%)	642 (10.2%)
Other (dementia, liver disease, AIDS)	829 (5.9%)	308 (4.9%)
Mean follow-up in years	6.3 (4.7)	5.6 (4.4)
Number of postoperative periprosthetic fractures during follow-up	305 (2.2%)	330 (5.3%)

**^a^**ASA: American Society of Anesthesiologists.

There were 305 postoperative periprosthetic fractures in the primary THR cohort and 330 in the revision THR cohort at postoperative day 1 onwards. The timing of these fractures in the primary and revision THR cohorts was as follows: day 1–30: 8% and 4%; day 31–90: 13% and 13%; day 91–365: 12% and 16%; and after day 365: 67% and 66%, respectively.

### Risk factors for periprosthetic fractures after primary THR

In crude (or unadjusted) analyses, several comorbidities (heart disease, peripheral vascular disease, renal disease, peptic ulcer disease, connective tissue disease, cancer, and other comorbidity (dementia, liver disease, AIDS)) were associated with higher risk of postoperative periprosthetic fractures after primary THR (Table 2). After simultaneously adjusting the analysis for other important factors (age, sex, BMI, implant fixation, ASA class, operative diagnosis) and all comorbidities in multivariable-adjusted analysis, we found that the presence of peptic ulcer disease was associated with 1.5 times higher hazard ratio and heart disease was associated with 1.7 times higher risk of periprosthetic fracture after primary THR, both statistically and clinically significantly (Table 2). Other comorbidities, significant in univariate analyses, were no longer independently associated with a higher risk in multivariable-adjusted analysis. Sensitivity analyses that adjusted for time period led to no (or minimal) change in the relationship of comorbidities with fracture risk (odds ratios changed minimally) and no change in statistical significance (Table 2). Time period was not significant in the model (p = 0.08), but showed a trend of decrease over time.

**Table 2.** Crude (or unadjusted) and multivariable-adjusted risk of postoperative periprosthetic fracture following primary total hip replacement (THR)

A	B	C	D	E	F
Heart disease (MI, CHF)			p < 0.001	p = 0.006	p = 0.006
No	12,854	264 (2%)	1.00 (ref)	1.00 (ref)	1.00 (ref)
Yes	1,211	41 (3%)	1.92 (1.38–2.67)	1.68 (1.16–2.42)	1.68 (1.16–2.42)
Peripheral vascular disease			p = 0.011	p = 0.1	p = 0.1
No	13,257	280 (2%)	1.00 (ref)	1.00 (ref)	1.00 (ref)
Yes	808	25 (3%)	1.70 (1.13–2.56)	1.42 (0.91–2.19)	1.42 (0.91–2.20)
Cerebrovascular disease, hemiplegia or paraplegia			p = 0.7	p = 0.2	p = 0.2
No	12,940	282 (2%)	1.00 (ref)	1.00 (ref)	1.00 (ref)
Yes	1,125	23 (2%)	1.08 (0.71–1.66)	0.75 (0.48–1.18)	0.74 (0.47–1.17)
Moderate-to-severe renal disease			p = 0.003	p = 0.4	p = 0.5
No	13,176	278 (2%)	1.00 (ref)	1.00 (ref)	1.00 (ref)
Yes	889	27 (3%)	1.81 (1.22–2.69)	1.20 (0.78–1.85)	1.16 (0.75–1.79)
Peptic ulcer disease			p < 0.001	p = 0.02	p = 0.02
No	13,034	267 (2%)	1.00 (ref)	1.00 (ref)	1.00 (ref)
Yes	1,031	38 (4%)	1.85 (1.32–2.60)	1.51 (1.06–2.15)	1.52 (1.07–2.17)
Chronic obstructive pulmonary disease (COPD)			p = 0.06	p = 0.5	p = 0.6
No	12,497	264 (2%)	1.00 (ref)	1.00 (ref)	1.00 (ref)
Yes	1,568	41 (3%)	1.37 (0.98–1.90)	1.12 (0.79–1.57)	1.10 (0.78–1.56)
Diabetes (with or without organ damage)			p = 0.1	p = 0.7	p = 0.8
No	12,830	276 (2%)	1.00 (ref)	1.00 (ref)	1.00 (ref)
Yes	1,235	29 (2%)	1.33 (0.91–1.95)	1.08 (0.72–1.61)	1.07 (0.71–1.60)
Other (dementia, liver disease, AIDS)			p < 0.001	p = 0.7	p = 0.07
No	13,236	275 (2%)	1.00 (ref)	1.00 (ref)	1.00 (ref)
Yes	829	30 (4%)	1.95 (1.34–2.85)	1.48 (0.97–2.25)	1.47 (0.97–2.25)
Connective tissue disease			p = 0.007	p = 0.5	p = 0.5
No	13,045	270 (2%)	1.00 (ref)	1.00 (ref)	1.00 (ref)
Yes	1,020	35 (3%)	1.62 (1.14–2.30)	1.15 (0.75–1.76)	1.15 (0.75–1.76)
Cancer			p = 0.003	p = 0.1	p = 0.1
No	11,899	243 (2%)	1.00 (ref)	1.00 (ref)	1.00 (ref)
Yes	2,166	62 (3%)	1.52 (1.15–2.00)	1.29 (0.94–1.77)	1.28 (0.93–1.75)

AVariable ASA: American Society of Anesthesiologists; MI: myocardial infarction; CHF: congestive heart failure. BTotal (n = 14,065) CPeriprosthetic fractures(n = 305) DUnivariate hazard ratio (95% CI) EMultivariable hazard ratio (95% CI)adjusted for age, gender, BMI, operative diagnosis, ASA class, implant fixation (cemented/hybrid, not cemented) and each Charlson comorbidity FMultivariable hazard ratio (95% CI) additionally adjusted for the year of surgery. Correlation coefficient between ASA and Charlson index was 0.33.

### Risk factors for periprosthetic fracture after revision THR

In crude (or unadjusted) analyses, peptic ulcer disease, heart disease, and connective tissue disease (rheumatological disorders) were each found to be associated with a higher risk of postoperative periprosthetic fracture (Table 3). In multivariable-adjusted analysis, we found that the presence of peptic ulcer disease was associated with significantly increased risk of periprosthetic fracture after revision THR, with a hazard ratio of 1.6 (Table 3). Other comorbidities were no longer independently associated with a higher risk in multivariable analyses. In sensitivity analyses that additionally adjusted the regression analyses for time period, we found no or minimal change in odds ratios and no change in statistical significance of association of peptic ulcer disease with fracture (Table 3). Time period was significant in the model (p = 0.01), showing a decrease in periprosthetic fractures after revision THR over time.

**Table 3.** Crude (or unadjusted) and multivariable-adjusted hazard of postoperative periprosthetic fracture following revision total hip replacement (THR)

A	B	C	D	E	F
Heart disease (MI, CHF)			p = 0.04	p = 0.1	p = 0.1
No	5,441	298 (6%)	1.00 (ref)	1.00 (ref)	1.00 (ref)
Yes	510	32 (6%)	1.47 (1.02–2.12)	1.39 (0.92–2.09)	1.38 (0.91–2.08)
Peripheral vascular disease			p = 0.2	p = 0.2	p = 0.3
No	5,670	313 (6%)	1.00 (ref)	1.00 (ref)	1.00 (ref)
Yes	281	17 (6%)	1.35 (0.83–2.21)	1.37 (0.82–2.29)	1.32 (0.79–2.22)
Cerebrovascular disease, hemiplegia or paraplegia			p = 0.2	p = 0.2	p = 0.3
No	5,543	306 (6%)	1.00 (ref)	1.00 (ref)	1.00 (ref)
Yes	408	24 (6%)	1.35 (0.89–2.05)	1.30 (0.84–2.02)	1.27 (0.82–1.98)
Moderate-to-severe renal disease			p = 0.2	p = 0.6	p = 0.8
No	5,585	308 (6%)	1.00 (ref)	1.00 (ref)	1.00 (ref)
Yes	366	22 (6%)	1.38 (0.89–2.13)	1.12 (0.70–1.78)	1.08 (0.67–1.72)
Peptic ulcer disease			p = 0.003	p = 0.02	p = 0.01
No	5,551	295 (5%)	1.00 (ref)	1.00 (ref)	1.00 (ref)
Yes	400	35 (9%)	1.69 (1.19–2.40)	1.58 (1.10–2.29)	1.61 (1.11–2.33)
Chronic obstructive pulmonary disease (COPD)			p = 0.6	p = 0.7	p = 0.7
No	5,416	301 (6%)	1.00 (ref)	1.00 (ref)	1.00 (ref)
Yes	535	29 (5%)	1.10 (0.75–1.61)	1.08 (0.73–1.60),	1.08 (0.72–1.60)
Diabetes (with or without organ damage)			p = 0.1	p = 0.2	p = 0.2
No	5,407	297 (5%)	1.00 (ref)	1.00 (ref)	1.00 (ref)
Yes	544	33 (6%)	1.38 (0.96–1.98)	1.32 (0.90–1.95)	1.31 (0.89–1.92)
Other (dementia, liver disease, AIDS)			p = 0.2	p = 0.7	p = 0.7
No	5,661	312 (6%)	1.00 (ref)	1.00 (ref)	1.00 (ref)
Yes	290	18 (6%)	1.41 (0.88–2.27)	1.10 (0.66–1.84)	1.09 (0.66–1.82)
Connective tissue disease			p = 0.02	p = 0.6	p = 0.6
No	5,393	285 (5%)	1.00 (ref)	1.00 (ref)	1.00 (ref)
Yes	558	45 (8%)	1.44 (1.05–1.97)	1.09 (0.78–1.53)	1.10 (0.79–1.54)
Cancer			p = 0.1	p = 0.09	p = 0.1
No	5,347	292 (5%)	1.00 (ref)	1.00 (ref)	1.00 (ref)
Yes	604	38 (6%)	1.31 (0.93–1.83)	1.37 (0.95–1.99)	1.36 (0.94–1.97)

AVariable (See Table 2) BTotal (n = 6,281) CPeriprosthetic fractures (n = 330) DUnivariate hazard ratio (95% CI) EMultivariable hazard **^a^** ratio (95% CI) adjusted for age, gender, BMI, operative diagnosis, ASA class, and each Charlson comorbidity. FMultivariable hazard **^b^** ratio (95% CI) additionally adjusted for the year of surgery.

### Periprosthetic fracture free survival after primary and revision THR

Using Kaplan-Meier curves, we examined the fracture-free survival by significant factors for primary and revision THR. Fracture-free survival was 99.2% at 1 year after primary THR and 98.1% at 1 year after revision THR (Table 4).

**Table 4.** Fracture-free survival, by significant factors, in patients with primary or revision THR. Values are percentages

Fracture-free survival rate at				
Variable	1 year	2 years	5 years	10 years
*Primary THR*				
Overall	99.2 (99.1–99.4)	98.9 (98.4–99.4)	98.6 (98.4–98.8)	97.0 (96.6–97.4)
Heart disease (MI, CHF)				
No	99.3 (99.1–99.4)	99.1 (98.9–99.2)	98.6 (98.4–98.8)	97.2 (96.8–97.6)
Yes	99.1 (98.5–99.6)	98.6 (97.9–99.3)	98.0 (97.2–98.9)	94.2 (92.0–96.3)
Peptic ulcer disease				
No	99.3 (99.1–99.4)	99.1 (98.9–99.3)	98.7 (98.4–98.9)	97.2 (96.8–97.6)
Yes	98.6 (97.8–99.3)	98.0 (97.1–98.9)	97.4 (96.3–98.5)	94.1 (92.0–96.3)
*Revision THR*				
Overall	98.1 (97.7–98.4)	97.4 (97.0–97.8)	95.6 (95.1–96.2)	92.0 (91.0–93.0)
Heart disease (MI, CHF)				
No	98.1 (97.8–98.5)	97.5 (97.1–97.9)	95.8 (95.2–96.4)	92.3 (91.3–93.3)
Yes	97.3 (95.8–98.8)	96.8 (95.2–98.4)	93.8 (91.3–96.4)	87.2 (81.7–93.2)

## Discussion

To our knowledge, there have been no studies published that have examined peptic ulcer disease and heart disease as risk factors for periprosthetic fracture in THR patients. Our observation of an association between peptic ulcer disease and periprosthetic fractures in patients with THR is interesting in light of (1) recently described associations between the medication used for treatment for peptic ulcer disease and the risk of osteoporotic fractures in general populations (Laine 2009) and (2) our recent observation of association of peptid ulcer disease with periprosthetic fractures after primary TKR (Singh and Lewallen 2011). The magnitude of increase in risk of periprosthetic fracture was similar for primary and revision THR, at about 50%, supporting the robustness of this association. Also, our finding of this association in three different cohorts (primary TKR, primary THR, and revision THR) indicates that this is a true association.

A recent editorial identified 3 key studies that provided evidence of increased fracture risk with the use of proton-pump inhibitors (Richards and Goltzman 2008). Both the dose and the duration of proton pump inhibitors were associated with higher risk of hip fracture in patients in the General Practitioners’ Research Database (Yang et al. 2006). Use of a proton-pump inhibitor for 7 or more years was associated with double the risk of osteoporotic fracture and a 5-times higher risk of hip fracture, and use for 5 or more years was associated with double the risk of hip fracture (Targownik et al. 2008). Another study found that use of a proton-pump inhibitor in the previous year was associated with increased fracture risk (18%, 45%, and 60% higher odds for overall fracture, hip fracture, and spine fracture risk, respectively) (Vestergaard et al. 2006). It has been hypothesized in previous studies that this effect may be mediated by lower calcium absorption due to hypochlorhydria related to proton-pump inhibitors. On the other hand, use of histamine-2 receptor antagonists was associated with increased fracture risk in one study (Yang et al. 2006), but reduced fracture risk in another study (Vestergaard et al. 2006). Due to the lack of availability of data on use of medication prior to and after THR in the Total Joint Registry, we were unable to test the hypotheses of whether the observed association was due to the disease or whether it was due to one of its treatments (such as use of proton-pump inhibitor).

The association between heart disease and periprosthetic fractures in patients with primary THR adds to our current knowledge. Recent publications have reported significant associations between cardiovascular disease and lower bone mineral density in the NHANES sample (Broussard and Magnus 2008) and between beta-blockers and fragility fractures in postmenopausal women (Sosa et al. 2011). Other authors have reported an association between postoperative use of statin and lower risk of revision (Thillemann et al. 2010), and between postoperative loop diuretic use and higher risk of revision due to deep infection and periprosthetic fracture (Thillemann et al. 2009), which raises questions related to the underlying mechanisms of the association between heart disease and periprosthetic fractures in the present study. Are these associations in this study related to the medications used for treatment of peptic ulcer disease and heart conditions? If so, which ones? Is the risk related to disease or to its severity? One recent genetic study in twins provided clues to the link between cardiovascular disease and hip fracture risk (Sennerby et al. 2009). People with heart disease had a higher risk of subsequent hip fracture. Increased risks in co-twins without an index diagnosis suggested that genetic factors may have a role in the association between cardiovascular disease and osteoporotic fractures. Our findings are in agreement with key findings from these earlier studies in larger cohorts that were not limited to arthroplasty.

The present study also provides information on the frequency of comorbidities in patients undergoing primary or revision THR. Patients had a mean of 1 comorbid condition in either cohort. This is not surprising, considering that the mean age of both cohorts was about 65 years.

We also observed that point estimates for several other comorbidities were similar to those for peptic ulcer disease, but not statistically significantly. For example, peripheral vascular disease and other diseases in primary THR cohort did not reach statistically significant levels of association with the fracture risk. Similarly, peripheral vascular disease, heart disease, and cancer in revision THR cohort did not reach statistically significance.

The study has some limitations. As with other registry studies, some patients may have been lost to follow-up—despite the close follow-up in our joint registry (visit to clinic, mailed questionnaire, telephone follow-up)—which would be expected to lead to underestimation of fractures. Thus, actual estimates of periprosthetic fractures may be higher. In addition, patients with higher comorbidity are also more likely to die during the follow-up, which reduces their risk of having a fracture, with death as the competing risk.

Censoring of observations at the time of death or fracture ensured that time for which a patient was observed in this study was only for those at risk of fracture (i.e. living patients at risk). Since the cohort grew over several decades and follow-up information was obtained through multiple sources, not just clinic visits, we were unable to calculate actual loss to follow-up. The Mayo Clinic provides both primary and specialty care to the local population and tertiary specialty care to those referred for THR; thus, generalization of these findings to all settings may not be possible. Residual confounding is possibly related to study design (non-randomized), although we attempted to control for several important variables. The strengths of our study include the large sample size, the use of prospective data from an institutional total joint registry, the ability to control for several important variables (BMI, ASA class, implant fixation), and the use of multivariable-adjusted estimates. Our estimates were quite robust, in that the associations were similar in the 2 cohorts (primary THR and revision THR) and the estimates for the significant associations were minimally attenuated after multivariable adjustment.
